# Znf76 is associated with development of the eyes, midbrain, MHB, and hindbrain in zebrafish embryos

**DOI:** 10.1080/19768354.2018.1557744

**Published:** 2019-02-22

**Authors:** Jangham Jung, S. Udhaya Kumar, Issac Choi, Tea-Lin Huh, Myungchull Rhee

**Affiliations:** a Department of Bioscience and Biotechnology, Graduate School, Chungnam National University, Daejeon, South Korea; b School of Life Sciences and Biotechnology, College of Natural Sciences, Kyungpook National University, Daegu, South Korea

**Keywords:** Znf76, MHB (Midbrain-hindbrain boundary), FGF signaling, Hindbrain, Eye

## Abstract

ZNF76 is a transcriptional repressor that targets the TATA-binding protein (TBP) and plays an essential role during brain development; however, its function during embryogenesis remains unclear. Here, we report the expression pattern and potential functions of *znf76* in zebrafish embryos. Maternal transcripts of *znf76* were detected at low levels in embryos at the 1-cell stage, with zygotic transcripts appearing at the sphere stage. At the bud stage, the distribution of *znf76* transcripts was polarized to the anterior and posterior regions of the embryos, and *znf76* transcripts were further restricted to the trigeminal placode and proctodeum posterior gut of the embryos at 18 h postfertilization (hpf). *znf76* transcripts were localized to the midbrain–hindbrain boundary (MHB), hindbrain, and developing eyes at 24 hpf. Ectopic expression of *znf76* with 5’-capped *znf76* mRNA microinjected into embryos at the 1-cell stage caused phenotypic defects in the eyes, MHB, hindbrain, and spinal cord. Overexpression of *znf76* resulted in a drastic reduction of *pax2a*, *fgf8a*, and *rx1* transcripts in the optic stalk, MHB, and eyes, respectively. Taken together, these data indicate that Znf76 governs developmental processes in the MHB, hindbrain, and eyes in zebrafish embryos. We also discuss the Fgf8 signaling networks associated with the Znf76 function.

## Introduction

Zinc finger proteins conduct a plethora of molecular functions, and contain one or more zinc atoms that play a major role in stabilizing the protein (Laity et al. 2001). In most species, the Cys_2_His_2_ (C_2_H_2_) domains serve as a transcription factor and recognize specific DNA sequences for gene regulation; zinc finger proteins with these domains are commonly known as the DNA-binding group (Laity et al. 2001). These C_2_H_2_ residues are highly conserved among vertebrates and contain 9 repeats of 30 cysteine and histidine amino acids (Brown et al. 1985; Miller et al. 1985). The family of zinc finger proteins can be classified into different fold groups, including C_2_H_2_-like, gag knuckle, treble clef, zinc ribbon, Zn2/Cys6, and TAZ2 domain-like, which, together with zinc-binding loops, confer folding and stability (Krishna et al. 2003). Zinc finger protein 76 (ZNF76) is a general transcription repressor targeting the TATA-binding protein (TBP) through a process regulated by sumoylation (Zheng and Yang 2004). ZNF76 is acetylated by p300 and deacetylated by histone deacetylase 1. Acetylation of ZNF76 leads to desumoylation of ZNF76, which attenuates its interaction with TBP (Zheng and Yang 2006). There are several studies that support an essential functional role for ZNF76 in central nervous system development (Armstrong et al. 1995; Kim et al. 1997). Mutagenesis studies *in vivo* have shown that some zinc finger proteins function as key regulators in the specification of dopaminergic and serotonergic neuronal cell fate (Dittrich et al. 1997; Lundell and Hirsh 1998; Guo et al. 2000). However, the potential embryological functions of ZNF76 have not yet been examined in vertebrate animal models.

Here, we use zebrafish as an animal model to address the function of ZNF76 in vertebrate embryonic development. We report for the first time the spatiotemporal expression patterns of zebrafish *znf76* and outline the molecular networks that may be associated with the functions of Znf76 in zebrafish embryos.

## Materials and methods

### Znf76 sequence analysis

Phylogenetic tree analysis was executed to investigate the evolutionary relationship between zebrafish Znf76 (NP_001071078.1) with human ZNF76 (NP_003418.2), chimpanzee ZNF76 (XP_001172167.1), mouse ZNF76 (NP_766205.1), chicken ZNF76 (XP_004935082.1), and *Xenopus* Znf76 (XP_017948566.1). Neighbor-joining method was applied for the phylogenetic tree, which was generated with the online available software, MEGA7.0 (https://www.megasoftware.net). Information about amino acids sequence for human, chimpanzee, mouse, chicken, *Xenopus*, and zebrafish was collected from the available proteins in NCBI, https://www.ncbi.nlm.nih.gov/protein/.

### Zebrafish maintenance and embryo generation

Wild type synchronized embryos were collected by natural breeding and maintained at 28.8°C. Zebrafish adult fishes and embryos were stored in the cycle of 10-hour dark/14-hour light and morphological confirmation was confirmed as mentioned in Kimmel et al. 1995. Phenylthiourea (PTU) with 0.2 mM concentration was introduced after passing 9 hpf to stop melanogenesis in the embryos.

### RNA preparation, cDNA synthesis, and RT–PCR

R&A-BLUE^TM^ Total RNA Extraction kit (iNtRON Biotechnology) was applied to isolate total RNA from various stages of zebrafish embryos and cDNA was synthesized with M-MLV Reverse Transcriptase (Enzynomics) with Oligo (dT)_20_ primer with the collected total RNA (Kumar et al. 2017). Designed oligos were employed to amplify the zebrafish *znf76* specific template (237 bp) with PCR experiment from various stages of embryos Forward primer; 5’ CGA CAT CAT CAG CTC ACA CCT G 3’ and Reverse primer; 5’ GCC TCC TCT AAC GTC TGT TGA TC 3’. The *β-actin* (500 bp) oligos were used as an internal control for this experiment, Forward Primer; GAG GAG CAC CCC GTC CTG C and Reverse Primer; GAT GGC TGG AAC AGG GCC. Upon completion of the PCR reaction, every set of reaction was confirmed with running 1% agarose gel in TE buffer using gel electrophoresis. After cloning in pGEMT-easy vector and confirmation with the digestion process, plasmid construct was sent for sequencing to SolGent Co. Ltd. The protocols were established and applied as previously in our laboratory (Kumar et al. 2017).

### Whole-mount *in situ* hybridization (WISH)

After confirming the sequences in the plasmid construct, cloned *znf76* construct was linearized with restriction enzyme and DIG-labeled anti-sense probe was synthesized with the RNA polymerase and sense probe was also synthesized as well to provide WISH in a negative manner, protocol was adapted as instructed by the Roche^TM^. Fixation of the embryos were performed with 4% paraformaldehyde (PFA) overnight at 4°C. To perform the WISH analysis, embryos over the 24 hpf were treated with the proteinase K in a respective time point as mention in laboratory protocols (Annupalle et al. 2017, Thisse et al. 2001). Images were captured when embryos were in 90% glycerol in PBST solution with Leica MZ16 (Kumar et al. 2017).

### Overexpression of *znf76* mRNA

Positive strand was selected for the *znf76* ORF (1551 bp) and primers were designed as; Forward Primer; ATA TGG AGG GGC TGG GGC TTC A, Reverse Primer; ATC ACT GAT CTG AGG TCA GTC CA. After completing the amplification, *znf76* cloning was performed in pcGlobin2 vector (Ro et al. 2004). Sequencing of the plasmid construct confirmed the insert in the pcGlobin2 vector. Linearization of the plasmid construct was done before synthesizing *znf76* capped mRNA with mMessage mMachine® High Yield Capped RNA Transcription Kit (Ambion® Applied Biosystems) and purified capped mRNA was injected (50pg and 100pg) in 1-cell stage of zebrafish embryos, phenol red dye with distilled water was injected as vehicle control in similar volume. Observation of the phenotypes of each embryos was done in every 6 h and images were taken at 24 hpf of the embryos.

## Results

### Zebrafish *znf76* encodes a novel C_2_H_2_ Zn finger protein

**Figure 1.**
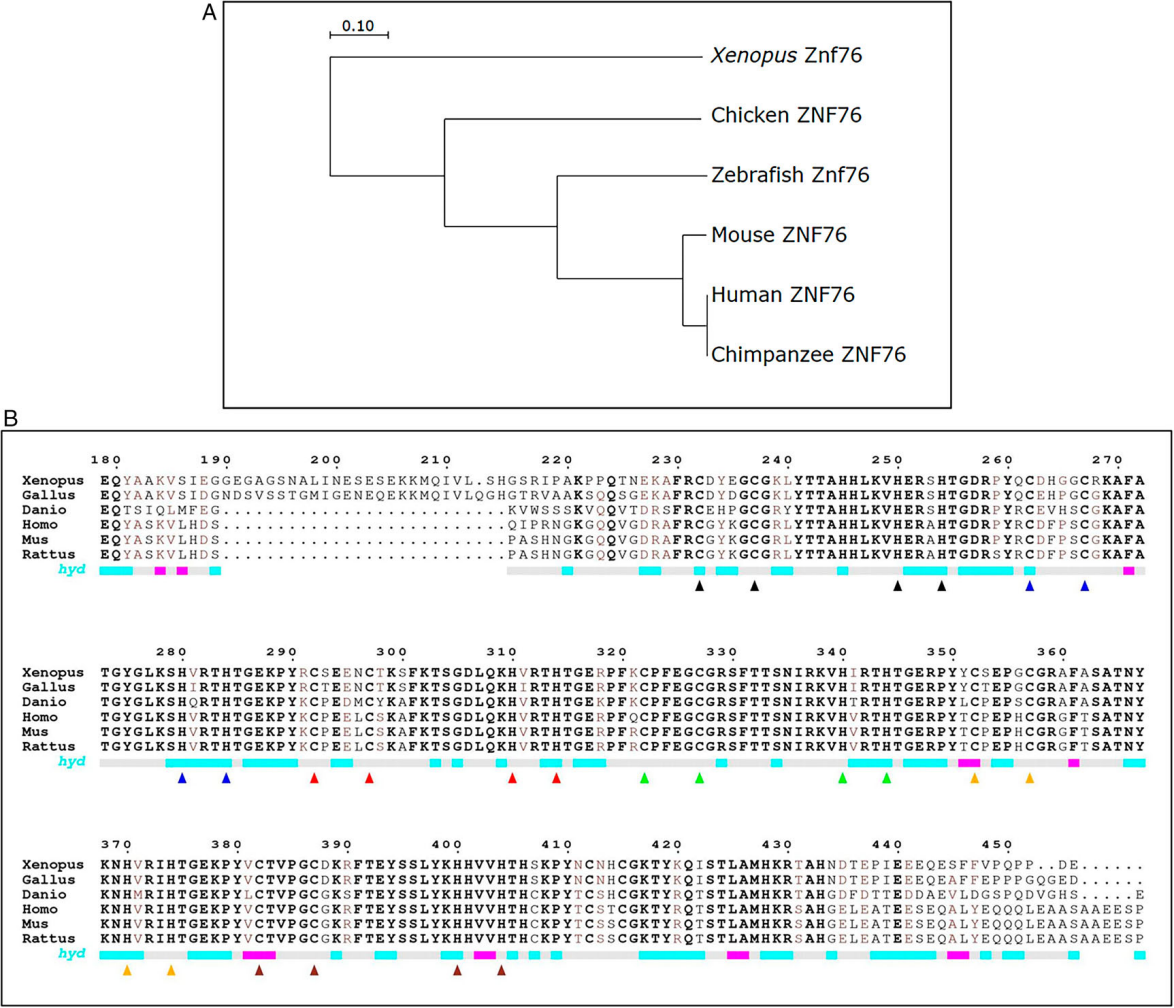
**(A) Phylogenetic analysis of Znf76 in higher vertebrates** The neighbor-joining method of MEGA7.0 was used to derive the phylogenetic tree for the amino acid sequences of human (NP_003418.2), chimpanzee (XP_001172167.1), mouse (NP_766205.1), chicken (XP_004935082.1), Xenopus (XP_017948566.1), and zebrafish (NP_001071078.1) Znf76, which were collected from the NCBI database. The scale bar represents branch distance. (**B) Homology analysis** The CLUSTAL OMEGA multiple sequence alignment tool was used to align the amino acid sequences of Znf76 from the zebrafish and higher animals. The alignment shows extensive similarity between zebrafish Znf76 and homologs in other vertebrates, suggesting that six C_2_H_2_ domains (triangle shapes) are evolutionarily conserved in vertebrate development.

We performed a bioinformatic search to identify zebrafish Znf76 (NCBI Refseq: NP_001071078.1), which is an ortholog of human ZNF76 (NCBI Refseq: NP_003418.2). We also compared the nucleotide sequences of *znf76* from the ENSEMBL (ID: ENSDARG00000013279) and NCBI GenBank (CU855848.15) databases to confirm its genomic organization (Nt 699,488–715,669). *znf76* is located on linkage group (LG) 22 in zebrafish and LG 6 in humans. Zebrafish Znf76 is composed of 516 amino acids. To assess whether zebrafish Znf76 is homologous with the protein in other vertebrates, we performed amino acid sequence alignment using Clustal Omega (https://www.ebi.ac.uk/Tools/msa/clustalo/) and plotted the results with Espript 3.0^4^ (http://espript.ibcp.fr) (Goujon et al. 2010; Sievers et al. 2011; McWilliam et al. 2013). At the protein level, zebrafish Znf76 shares 59% sequence similarity with human ZNF76. Multiple sequence alignment of zebrafish Znf76 shows that the C_2_H_2_ domain is highly conserved among vertebrates (Figure 1(A)) and that the protein shares approximately 59–60% amino acid sequence similarity with *Homo sapiens* (NP_003418.2), *Mus musculus* (NP_766205.1), *Rattus norvegicus* (NP_001128227.1), *Gallus gallus* (XP_426401.3), and *Xenopus tropicalis* (XP_017948566.1) (Figure 1(B)). These data demonstrate that zebrafish Znf76 is a homolog of human ZNF76.

### Spatiotemporal distribution of *znf76* transcripts during zebrafish embryogenesis

**Figure 2.**
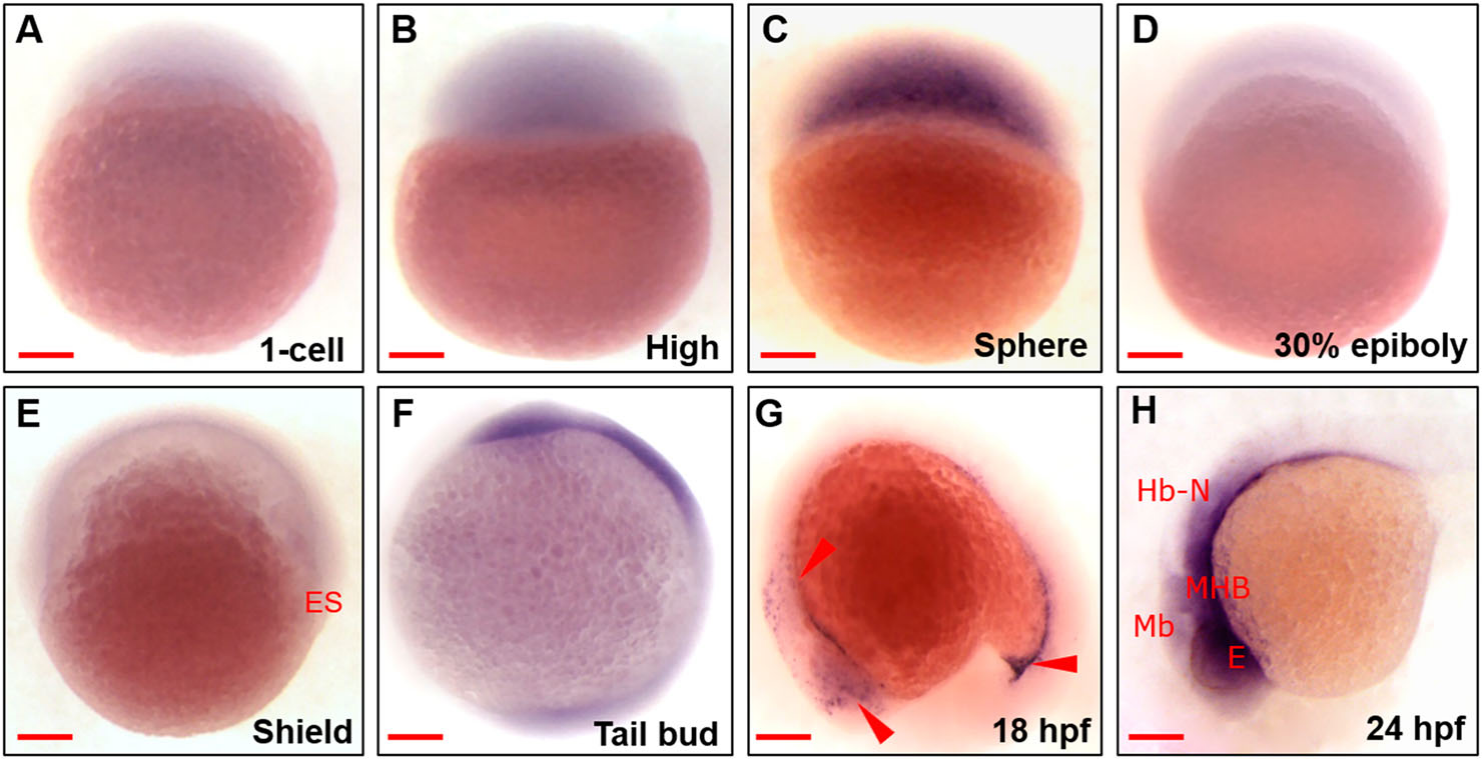
**Spatiotemporal expression of zebrafish *znf76* during embryonic development** WISH experiments performed with a *znf76* antisense RNA probe at the 1-cell (A), high (B), sphere (C), 30% epiboly (D), shield (E), tail bud (F), 18 hpf (G), and 24 hpf (H) stages. All the images are from the lateral view (A–H). *znf76* transcripts were zygotically expressed in the early stages and were reduced in the 30% epiboly and shield stages (D,E). *znf76* transcripts were present in the head and tail region (red arrowheads) at the tail bud stage (F) and then became progressively restricted to the trigeminal placode and proctodeum posterior gut, with a significant reduction in expression by the late somite stage (18 hpf) (G). At 24 hpf (H), *znf76* transcripts were restricted to Mb, MHB, and Hb-N. Red arrowheads show the anatomical structures. Mb – midbrain, MHB – midbrain–hindbrain boundary, Hb-N – hindbrain neurons, E – eye, ES – Embryonic shield. Scale bar: 100 µm.

To investigate the spatiotemporal expression patterns of *znf76* during zebrafish embryogenesis, we conducted whole-mount in situ hybridization (WISH) at various developmental stages using a *znf76*-specific antisense RNA probe. Maternal *znf76* transcripts were detected at low levels in embryos at the 1-cell stage (Figure 2(A)); zygotic transcripts were significantly increased at the sphere stage (Figure 2(C)). Distribution of *znf76* transcripts was polarized to the anterior and posterior regions at the bud stage (Figure 2(F)). *znf76* transcripts were further restricted to the trigeminal placode and proctodeum posterior gut at the late somite stage, 18 h postfertilization (hpf) (Figure 2(G)). At 24 hpf, they were specifically localized at the midbrain–hindbrain boundary (MHB) and in the hindbrain and developing eyes of the embryos (Figure 2(H)). These observations show that the distribution of *znf76* transcripts is modulated spatiotemporally and that *znf76* transcripts are restricted to the CNS during the later stages of embryonic development.

### Ectopic expression of *znf76* causes phenotypic defects in the eyes, MHB, hindbrain, and spinal cord

**Figure 3.**
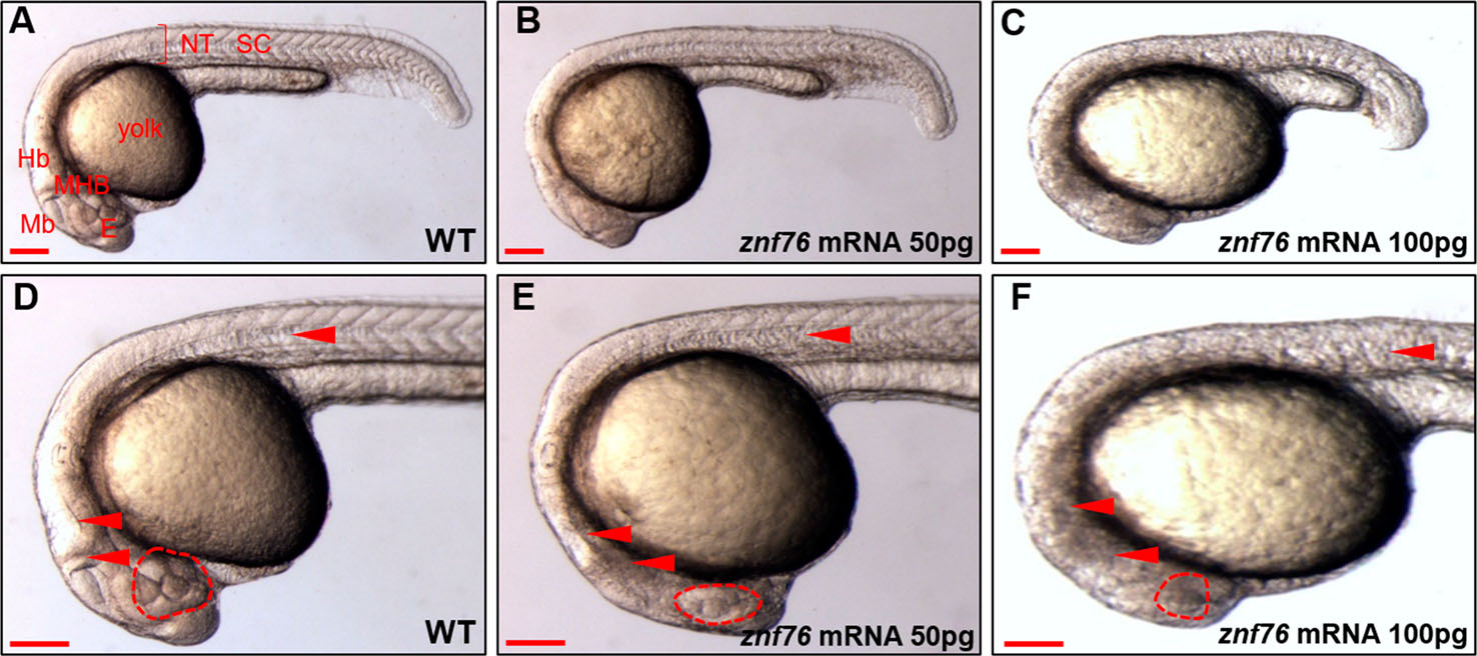
**Effects of ectopic expression of *znf76* mRNA in zebrafish embryos** Lateral view of the embryos (A–C) and magnified lateral view of the embryos (D–F) at 24 hpf. (A,D) WT zebrafish embryo injected with phenol red dye in distilled water, (B,E) zebrafish embryo injected with 50 pg of *znf76* mRNA, and (C,F) zebrafish embryo injected with 100 pg of znf76 mRNA. Microinjection of 50 or 100 pg of *znf76* mRNA was performed at the 1-cell stage to induce overexpression. Red arrowheads indicate the specific tissues altered at 24 hpf by 50 or 100 pg of *znf76* mRNA injection, compared with the WT; red dotted circles indicate the size of the eyes. NT – neural tube, SC – spinal cord, Hb – hindbrain, Mb – midbrain, E – eye, TB – tail bud, NT – neural tube. Scale bars 100 μm.

To investigate the functions of Znf76 in zebrafish embryogenesis, we induced ectopic expression of *znf76* by microinjecting *znf76* capped mRNA into zebrafish embryos at the 1- or 2-cell stage. Embryos microinjected with 50 pg of *znf76* mRNA showed significantly retarded epiboly formation (data not shown). When the *znf76* mRNA-injected embryos reached 24 hpf, they showed various detailed phenotypic defects such as smaller eyes, a reduction in brain size accompanying an obscure MHB, a shortened anteroposterior axis, and a twisted spinal cord (Figure 3(B,E)). A control group microinjected with phenol red dye diluted in distilled water had a normal phenotype (Figure 3(A,D)). When 100 pg of *znf76* mRNA was injected per embryo, the phenotypic defects became more extreme (Figure 3(C,F)). The chart in Supplementary Table 1 illustrates the severity of defects in the size of the eyes, classified into four groups: normal, intermediate, severe, and dead. The severity of the phenotypic defects was proportional to the amount of *znf76* mRNA microinjected. Taken together, these results suggest that overexpression of *znf76* disturbs the normal development of the eyes, MHB, hindbrain, and spinal cord.

### Marker-gene studies in zebrafish embryos overexpressing *znf76*

**Figure 4.**
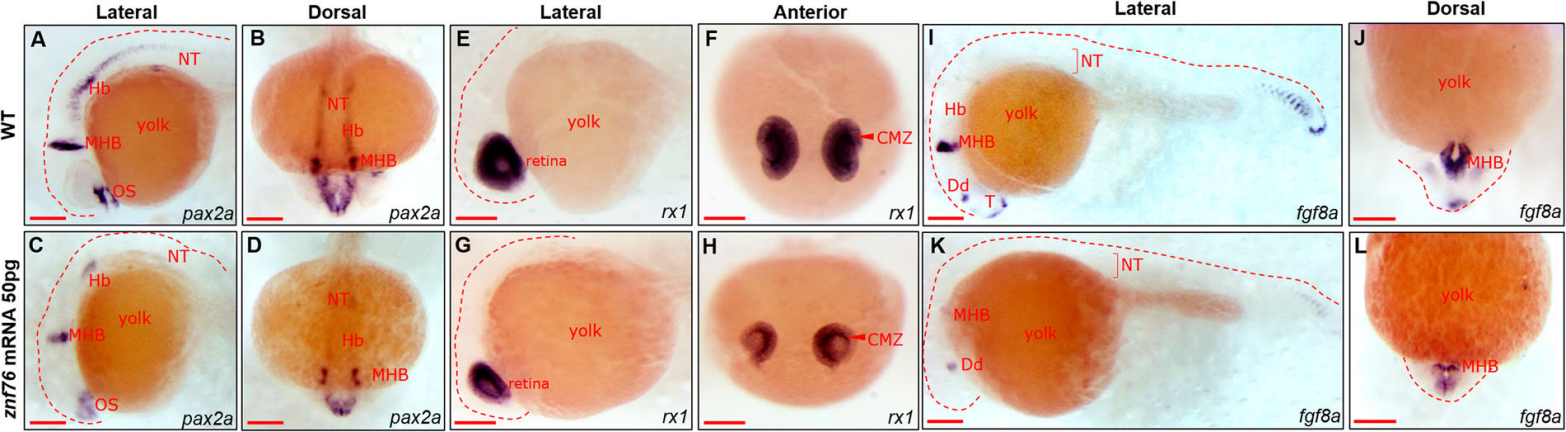
**Marker analysis in *znf76*-overexpressing embryos** WISH was performed at 24 hpf in znf76-overexpressing embryos with *pax2a*, *rx1*, and *fgf8a* to investigate the morphological defects at a molecular level. Lateral view (A, C, E, G, I, and K); anterior view (B, D, F, H, J, and L). *pax2a* transcripts were significantly reduced in the optic stalk, MHB, and hindbrain neurons compared with the WT (A-D). *rx1* marks neural cells in the retina of WT embryos at 24 hpf (E,F). *rx1* transcripts were reduced in *znf76*-overexpressing embryos in the retina, especially in the CMZ area of the eyes (F,H); the size of the eyes was also reduced compared with the WT (E,G). *fgf8a* is a marker for the dorsal diencephalon, MHB, posterior somites, and posterior notochord. At 24 hpf, there was a marked reduction in *fgf8a* transcripts in these regions in *znf76*-overexpressing embryos (K,L) compared with the WT (I,J). CMZ – central marginal zone, Dd – dorsal diencephalon, MHB – midbrain–hindbrain boundary, Hb – hindbrain, T – telencephalon, NT – neural tube, OS – optic stalk. Scale bar: 100 µm.

To identify the molecular events associated with the biological functions of Znf76, we used WISH to analyze embryos ectopically expressing *znf76*, using the molecular markers *pax2a*, *rx1*, and *fgf8a* (Krauss et al. 1991; Reifers et al. 1998; Chuang et al. 1999). *pax2a* is a transcription factor that controls the regulatory gene network that maintains MHB specification (Krauss et al. 1992) and is involved in the specification of the otic placode (Hans et al. 2004). At 24 hpf, ectopic expression of *znf76* markedly reduced both the level of *pax2a* transcripts and the area containing *pax2a* transcripts in the MHB, optic stalk, and hindbrain (Figure 4(C,D)), compared with the WT (Figure 4(A,B)). During development, cells in the retina differentiate, ultimately giving rise to the bipolar, photoreceptor, and ganglion cell layers (Chuang et al. 1999). Because ectopic expression of z*nf76* disturbed normal development of the eyes (Figure 3(E,F)), we used WISH to measure the levels of *rx1*, a molecular marker for the retina, in embryos overexpressing z*nf76*. In the control group, *rx1* transcripts were abundant and restricted to the neural retina at 24 hpf (Figure 4(E,F)), but in embryos ectopically expressing *znf76*, *rx1* transcripts were significantly reduced in the central marginal zone (CMZ) of the eyes (Figure 4(G,H)). *fgf8a* governs the organization of the midbrain and hindbrain by controlling the proliferation and differentiation of cells in these regions (Raible and Brand 2004; Harris et al. 2011). In our WISH analysis, *fgf8a* transcripts were significantly reduced at 24 hpf in the midbrain and hindbrain regions of embryos ectopically expressing *znf76* (Figure 4(K,L)) compared with the WT (Figure 4(I,J)). These observations support the hypothesis that *znf76* is associated with proper development and differentiation of the MHB, midbrain, hindbrain, and eyes in zebrafish embryogenesis.

## Discussion

The spatiotemporal expression pattern of *znf76* in normally developing embryos and the phenotypic defects induced by ectopic expression of *znf76* both strongly support the hypothesis that transcriptional regulation of *znf76* is important for proper development of the eyes, MHB, and hindbrain. We thus searched for *cis*-acting elements that might be present in the regulatory regions of *znf76*. As shown in Figure 4(K,L), *fgf8a* transcripts were almost completely absent at 24 hpf in the midbrain and hindbrain regions of embryos ectopically expressing z*nf76*. Fgf8a governs the organization of the midbrain and hindbrain (Raible and Brand 2004; Harris et al. 2011), and extracellular signal-related kinases (ERKs) are known to be involved in the downstream FGF8 signaling pathways (Harada et al. 2016). Therefore, we tested the inhibitory effects of ERKs on zebrafish embryonic development by applying two ERK inhibitors, UO126 for ERK1/2 and SB203580 for ERK3/4, which are frequently used to block ERK signaling pathways *in vitro* (Newton et al. 2000). Our preliminary data indicate that inhibition of ERK signaling in zebrafish embryos mimics the effects of overexpression of *znf76* in terms of the spatiotemporal expression patterns of *pax2a*, *rx1*, and *fgf8a* in embryos at 24 hpf (data not shown). Furthermore, measurements of the transcriptional activity of the *znf76* promoter region show that the two ERK inhibitors repress the transcription of *znf76* (data not shown). It is worth noting that ectopic expression of *znf76* altered the expression level and pattern of *pax3a* and *rx1* transcripts (Figure 4), but not of other molecular marker genes, such as *emx1* and *rx3* (Supplementary Figure 1). It is thus probable that Znf76 is associated with the development of the eyes, midbrain, MHB, and hindbrain in zebrafish embryos through gene-specific transcriptional regulation in response to ERK signaling pathways.

## Supplementary Material

Supplementary_Fig._1.tif
