# Supplementary figures and images for: Znf76 is associated with development of the eyes, midbrain, MHB, and hindbrain in zebrafish embryos
Source: Anim Cells Syst (Seoul). 2019 Feb 22;23(1):26–31. doi: 10.1080/19768354.2018.1557744 (PMC6394295; doi:10.1080/19768354.2018.1557744)

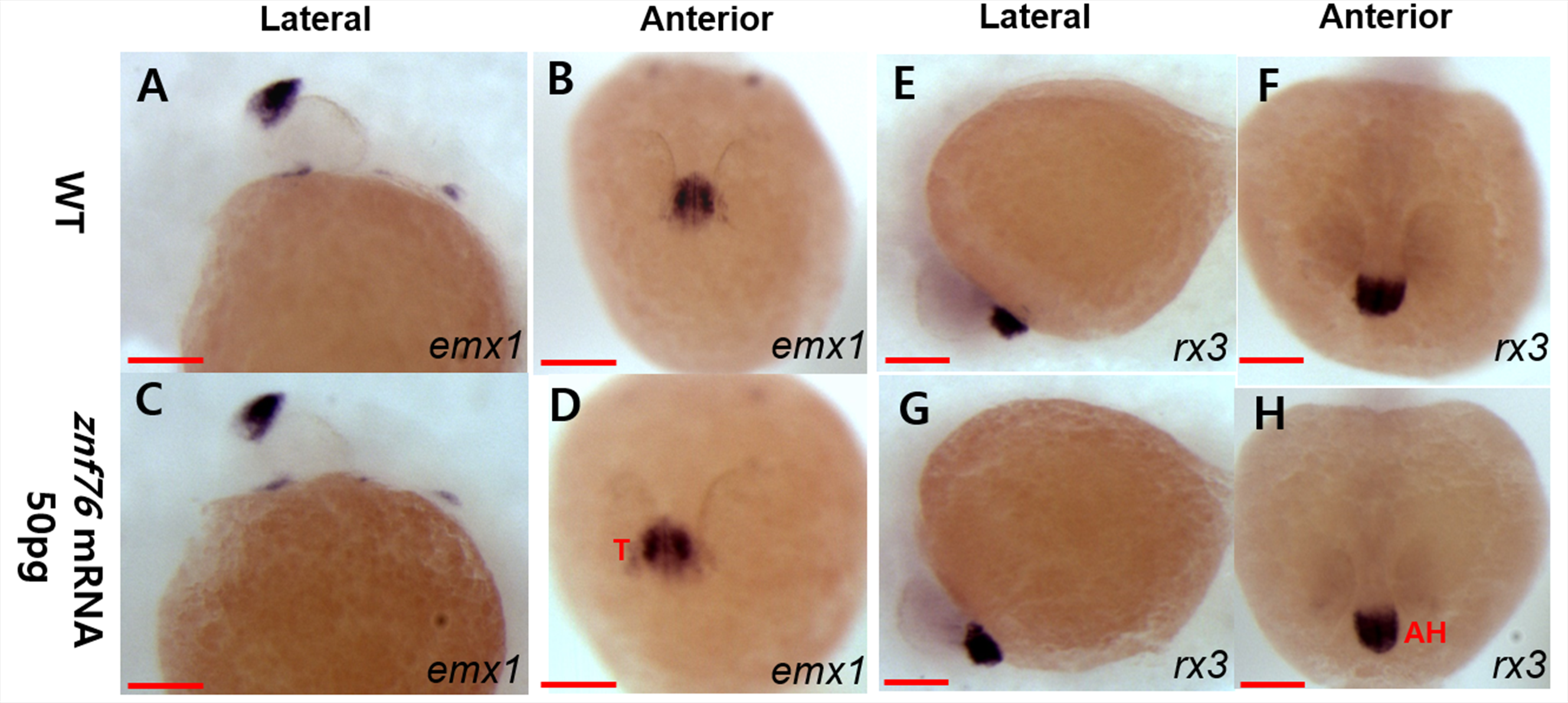

Supplement: Supplementary_Fig._1.tif [file TACS_A_1557744_SM2003.tif]
